# Evaluation of a PET Insert for Trimodal Imaging: A Step Toward PET/MRI-Guided Focused Ultrasound

**DOI:** 10.1109/trpms.2025.3615467

**Published:** 2025-09-29

**Authors:** F. Lopez-Berenguer, A. Gonzalez-Montoro, M. Freire, A. González-Tamarit, S. Jiménez-Serrano, L. F. Vidal, M. Gil, F. Loignon-Houle, I. Torres, J. L. Sachs, J. V. Rispoli, N. Tustison, S. S. Berr, M. B. Williams, D. Andrés, J. J. Rodríguez-García, J. L. Alonso-Ramos, N. Jiménez, A. Carrión, F. Camarena, T. Guallart-Naval, J. M. Algarín, R. Bosch, E. Pallás, J. Alonso, N. D. Sheybani, S. Maslova, T. Sherlock, A. Thede, Z. E. F. Demir, M. Hoch, J. He, J. M. Benlloch, M. J. Rodríguez-Álvarez, A. J. González

**Affiliations:** Detectors for Molecular Imaging Laboratory, Institute of Instruments for Molecular Imaging, 46022 Valencia, Spain.; Detectors for Molecular Imaging Laboratory, the Instituto de Instrumentación para Imagen Molecular, and the Centro Mixto CSIC, Universitat Politècnica de València, 46022 Valencia, Spain.; Detectors for Molecular Imaging Laboratory, the Instituto de Instrumentación para Imagen Molecular, and the Centro Mixto CSIC, Universitat Politècnica de València, 46022 Valencia, Spain.; Detectors for Molecular Imaging Laboratory, the Instituto de Instrumentación para Imagen Molecular, and the Centro Mixto CSIC, Universitat Politècnica de València, 46022 Valencia, Spain.; Detectors for Molecular Imaging Laboratory, the Instituto de Instrumentación para Imagen Molecular, and the Centro Mixto CSIC, Universitat Politècnica de València, 46022 Valencia, Spain.; Detectors for Molecular Imaging Laboratory, the Instituto de Instrumentación para Imagen Molecular, and the Centro Mixto CSIC, Universitat Politècnica de València, 46022 Valencia, Spain.; Detectors for Molecular Imaging Laboratory, the Instituto de Instrumentación para Imagen Molecular, and the Centro Mixto CSIC, Universitat Politècnica de València, 46022 Valencia, Spain.; Detectors for Molecular Imaging Laboratory, the Instituto de Instrumentación para Imagen Molecular, and the Centro Mixto CSIC, Universitat Politècnica de València, 46022 Valencia, Spain.; Department of Nuclear Medicine, Hospital La Fe, 46026 Valencia, Spain.; Department of Radiology and Medical Imaging, University of Virginia, Charlottesville, VA 22904 USA.; Department of Radiology and Medical Imaging, University of Virginia, Charlottesville, VA 22904 USA.; Department of Radiology and Medical Imaging, University of Virginia, Charlottesville, VA 22904 USA.; Department of Radiology and Medical Imaging, University of Virginia, Charlottesville, VA 22904 USA.; Department of Radiology and Medical Imaging, University of Virginia, Charlottesville, VA 22904 USA.; Centro Mixto CSIC and the Instituto de Instrumentación para Imagen Molecular, Universitat Politècnica de València, 46022 Valencia, Spain.; Centro Mixto CSIC and the Instituto de Instrumentación para Imagen Molecular, Universitat Politècnica de València, 46022 Valencia, Spain.; Centro Mixto CSIC and the Instituto de Instrumentación para Imagen Molecular, Universitat Politècnica de València, 46022 Valencia, Spain.; Centro Mixto CSIC and the Instituto de Instrumentación para Imagen Molecular, Universitat Politècnica de València, 46022 Valencia, Spain.; Centro Mixto CSIC and the Instituto de Instrumentación para Imagen Molecular, Universitat Politècnica de València, 46022 Valencia, Spain.; Centro Mixto CSIC and the Instituto de Instrumentación para Imagen Molecular, Universitat Politècnica de València, 46022 Valencia, Spain.; Centro Mixto CSIC and the Instituto de Instrumentación para Imagen Molecular, Universitat Politècnica de València, 46022 Valencia, Spain.; Centro Mixto CSIC and the Instituto de Instrumentación para Imagen Molecular, Universitat Politècnica de València, 46022 Valencia, Spain.; Centro Mixto CSIC and the Instituto de Instrumentación para Imagen Molecular, Universitat Politècnica de València, 46022 Valencia, Spain.; Centro Mixto CSIC and the Instituto de Instrumentación para Imagen Molecular, Universitat Politècnica de València, 46022 Valencia, Spain.; Centro Mixto CSIC and the Instituto de Instrumentación para Imagen Molecular, Universitat Politècnica de València, 46022 Valencia, Spain.; Biomedical Engineering Society, University of Virginia, Charlottesville, VA 22904 USA.; Biomedical Engineering Society, University of Virginia, Charlottesville, VA 22904 USA.; Biomedical Engineering Society, University of Virginia, Charlottesville, VA 22904 USA.; Biomedical Engineering Society, University of Virginia, Charlottesville, VA 22904 USA.; Biomedical Engineering Society, University of Virginia, Charlottesville, VA 22904 USA.; Biomedical Engineering Society, University of Virginia, Charlottesville, VA 22904 USA.; Department of Radiology and Medical Imaging, University of Virginia, Charlottesville, VA 22904 USA.; Centro Mixto CSIC and the Instituto de Instrumentación para Imagen Molecular, Universitat Politècnica de València, 46022 Valencia, Spain.; Centro Mixto CSIC and the Instituto de Instrumentación para Imagen Molecular, Universitat Politècnica de València, 46022 Valencia, Spain.; Detectors for Molecular Imaging Laboratory, the Instituto de Instrumentación para Imagen Molecular, and the Centro Mixto CSIC, Universitat Politècnica de València, 46022 Valencia, Spain.

**Keywords:** Focused ultrasound (FUS), magnetic resonance imaging (MRI), positron emission tomography (PET), trimodal imaging

## Abstract

Combining positron emission tomography (PET) with magnetic resonance imaging (MRI) and focused ultrasound (FUS) has emerged as a promising hybrid technique in the medical imaging field. Despite the potential benefits of using simultaneous PET and MRI acquisitions to monitor therapeutic effects and ensure precision and safety during FUS applications, no commercial or academic PET/MRI-guided FUS system (referred in the following as trimodal PET-MRI-FUS) exists. This work presents the design and evaluation of a preclinical PET insert for simultaneous operation with MRI and FUS systems. The proposed PET insert is based on monolithic LYSO crystals arranged in two octagonal rings that define inner and outer diameters of 72 and 114 mm, respectively, with an axial length of 67 mm. The system performance was evaluated according to the NEMA NU 4 2008 protocol. It achieves a uniform spatial resolution of ~0.9 mm along the whole PET Field of View (FOV) when enabling Depth of Interaction (DOI) capabilities. Moreover, we found a sensitivity peak of 3.8% for an energy window of 255–766 keV and a peak noise equivalent count rate peak of 80 kcps at an activity of 19 MBq. The compatibility of the PET insert with both low- and high-field MRI systems, as well as with custom and commercially available FUS devices, was studied using phantoms and in vivo experiments, respectively. The results of the trimodal tests have proven that the PET insert works simultaneously with both MRI and FUS systems.

## I. Introduction

Positron emission tomography (PET), magnetic resonance imaging (MRI), and focused ultrasound (FUS) are complementary techniques widely used in biomedical research. PET constitutes one of the leading molecular imaging techniques, enabling noninvasive and in vivo imaging of metabolic and functional biological processes [1]. MRI provides high-resolution anatomical imaging, offering detailed insights into tissue structures without ionizing radiation. Recently, FUS has gained interest as a noninvasive, repeatable, and safe interventional technique, capable of delivering therapeutic ultrasound energy to precise locations within the body. Applications include a diverse array of biological responses, from thermal to mechanical effects, within the targeted tissue [2]. Localized, transient blood-brain barrier opening (BBBO) induced by low-intensity, pulsed FUS, and microbubbles temporarily increases the permeability of brain vasculature, enhancing macromolecular payload delivery to and penetrance within naïve and pathologic brain tissue [3].

FUS BBBO—which is dictated by several sonication parameters, such as peak negative pressure, pulse duration, frequency, duty cycle, and burst length—requires precise spatial accuracy and real-time monitoring to prevent the potential tissue damage caused by overheating or excessive pressure. PET imaging can provide quantitative metabolic information to assess the biological effects of FUS, while MRI contributes high-resolution anatomical imaging and real-time thermometry, essential for guiding and monitoring the procedure [4]. The combination of these three techniques could enable more accurate and safe therapeutic applications, while providing both functional and structural information.

A previous study explored the use of sequential imaging methods, using MRI-guided FUS for BBBO and therapeutic agent delivery [5]. This study demonstrated that MR-guided FUS-induced BBBO improved the precision of targeting treatment areas, while PET provided longitudinal monitoring of CD47 antagonist delivery to the tumor. Despite their valuable contributions, these sequential systems face inherent limitations. The use of a sequential approach requires multiple steps, which prevents a comprehensive, real-time assessment of the tumor and hinders the ability to monitor drug delivery to the target area, complicating the optimization of therapies. This means that, despite the safely repeatable nature of FUS, insights are also presently limited for rationally informing repetition or adaptation of treatments. While there have been advances in integrating PET and MRI, as well as combining MRI and FUS, no system currently exists that simultaneously integrates all three. This gap is what our study seeks to address.

Combining PET, MRI, and FUS entails a unique set of challenges relative to conventional imaging setups [6]. PET scanners are not specifically designed for integration with high-field MRI, and image quality (IQ) often suffers from spatial resolution degradation and electromagnetic interference [7]. Additionally, the presence of a standard ultrasound probe inside a PET system can yield image artifacts due to attenuation and scatter of annihilation photons. These limitations have driven the development of specialized PET inserts that maintain high spatial resolution and sensitivity while ensuring compatibility with MRI and ultralight custom-designed ultrasound probes. One promising example of an integrated imaging system is the PETRUS system, which combines PET with ultrafast ultrasound imaging (UUI), enabling simultaneous imaging without significant degradation in PET IQ [8].

We have developed a preclinical PET insert specifically designed for simultaneous operation with MRI and FUS systems, enabling concurrent PET and MRI scanning during FUS treatments. This insert is based on monolithic scintillators coupled to silicon photomultipliers (SiPMs) that provide accurate 3-D gamma-ray position, including Depth of Interaction (DOI). This DOI information is used to reduce parallax errors, resulting in a homogeneous spatial resolution across the Field of View (FOV).

The system has been evaluated following a modified NEMA NU 4–2008 protocol [9] for assessing spatial resolution, sensitivity, count rate performance and IQ, in which maximum-likelihood expectation-maximization (MLEM) reconstruction was employed instead of filtered back projection (FBP) for spatial resolution assessment. Images of additional phantoms, including a micro-Derenzo and a mouse-like fillable phantom, were measured and evaluated to further study the imaging capabilities of the PET insert. The results of the evaluation have been compared with the ones reported for other preclinical PET inserts of similar dimensions: Aspect Imaging SimPET/M7 [10], MR Solutions I-402 [11], MADPET4 [12], and Bruker Si-198 [13].

To assess the performance and compatibility of the PET insert as part of a trimodal system, additional tests were conducted. In the first phase, the PET insert was tested for compatibility using a custom-designed phantom in combination with a low-field MRI and a custom-built FUS system. In the second phase, the PET insert was subjected to preliminary in vivo testing with a high-field MRI and a commercial FUS system (RK300; FUS Instruments).

## II. Materials and Methods

### A. PET System

**Fig. 1.**
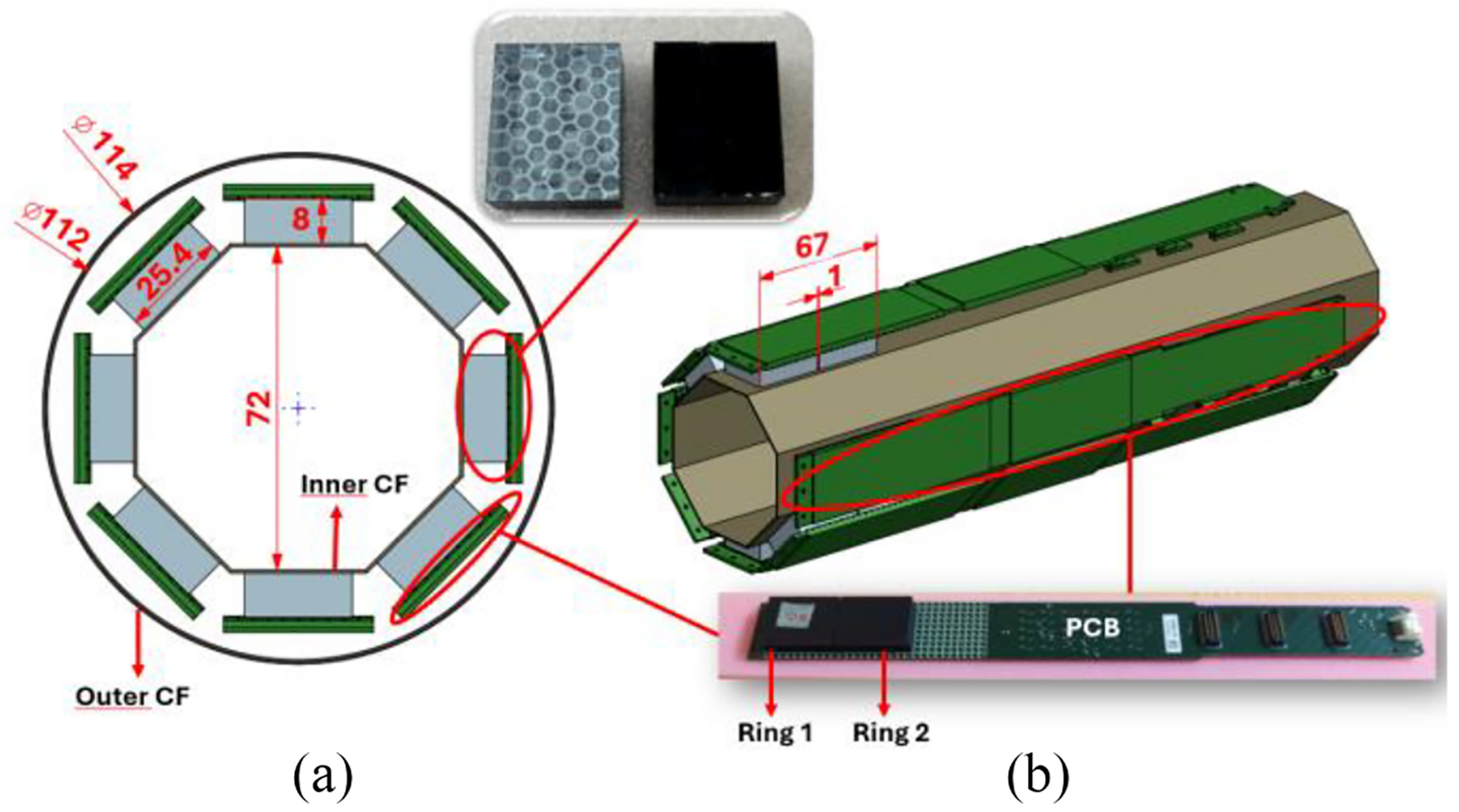
Sketch of the PET system with photographs of one crystal and one PCB. (a) Frontal view of the octagonal geometry of the PET insert. (b) Lateral view of the system. All dimensions are in mm.

The PET insert incorporates 16 Lu1.8Y2SiO5:Ce (LYSO:Ce) monolithic scintillators with dimensions of 3.0×25.4×8.0 mm. The scintillators are placed in a 2-ring octagonal structure, with eight crystals per ring and a gap of ~1 mm between rings. As shown in Fig. 1, this geometry defines inner and outer diameters of 72 and 114 mm, respectively, with axial and transaxial FOVs of 67 and 66 mm, respectively.

All crystal surfaces are polished, the lateral ones are painted black, and one of the 33.0×25.4 mm surfaces (referred as entrance face) includes a retroreflector layer to preserve the scintillation light distribution (LD) profile as much as possible [14]. The other 33.0×25.4 mm unpainted surface (referred as exit face) is coupled to the SiPM array using optical silicon (SYLGARD 184, Dow Corning, refractive index 1.4) [15]. Pairs of photosensor arrays are located on a printed circuit board (PCB) and consist of 8×10 SiPM elements (J-Series, OnSemi, 3×3 mm active area, 4.36-mm pitch) [16]. Regarding the readout electronics, each PCB includes a multiplexing circuitry that provides the projection of the SiPM rows and columns (8 + 10 output signals for each array) [17] and a temperature sensor. Note that since the SiPMs are sensitive to temperature variations, the temperature of the detectors was monitored and kept stable at 23 ± 1 °C using a refrigeration circuitry based on vortex tubes [18].

The output signals of each PCB are sent to the data acquisition (DAQ) system, which is composed of eight analog to digital converter (ADC) boards with 12-bit precision, a 250-ns integration window, and a custom-designed trigger board for coincidence acquisition.

Since the PET system was designed to work in the presence of magnetic fields, the PCBs were specifically designed without the use of ferromagnetic materials. This reduces the generation of eddy currents and the induction of noise in the electronic boards that are usually produced when switching the gradient fields. Moreover, the structure of the PET system was surrounded by a custom-designed Faraday cage made out of carbon fibers (CFs) to shield the MR radiofrequency field (RF) and to prevent RF electronic noise leakage into the MRI system [Fig. 1(a)] [19].

### B. Data Processing PET System

For the estimation of the *x* and *y* annihilation photon impact coordinates, the raise to a power (RTP) approach was used. This is a modified version of the Center of Gravity (CoG) algorithm in which the signal values are raised to a power of 2.0, prior to CoG calculation [20]. The deposited photon energy was estimated as the average of the summed projections from the 8 rows and 10 columns of SiPMs. The DOI coordinate was then estimated by averaging the ratio of these summed projections (E) to the maximum intensity value (Imax) from both rows and columns, using the E/Imax method [14].

In the case of monolithic-based PET detectors, the LD is truncated toward the border of the crystal, producing the so-called edge effects [14]. Other factors, such as variations in the optical coupling, manufacturing imperfections, and differences in photodetector response, also contribute to inaccuracies in the estimated 3-D photon impact position and energy. Therefore, an accurate calibration of all the detector blocks within the system is needed. For this, a Voronoi-based calibration methodology was implemented to calibrate both the 3-D impact position, including the DOI coordinate, and the deposited energy [21]. For this, with the modules already assembled in the PET system, experimental data was individually acquired for each detector using a custom 11 × 8 fillable well phantom (well pitch of 3 mm) attached to a tungsten mask with holes of 1 mm in diameter and 10-mm thick. Complementary to the physical collimation, software collimation was also applied [22] with an acceptance angle of 8 °. The calibration factors were extracted following the procedure explained in [21], obtaining a Look Up Table per module.

The DOI coordinate was used to calculate the true line of response before the reconstruction process and, thus, mitigate the so-called parallax error. Finally, a list-mode (LM) file, including all the coincidence events, was generated.

### C. Reconstruction

Image reconstruction was performed using the MLEM algorithm [23]. The MLEM algorithm was implemented in a custom-built framework optimized with GPU acceleration [24], [25]. An isotropic voxel size of 0.5 mm^3^ was employed. Normalization corrections and an energy window of 408–613 keV (20% of 511 keV) were applied for all the reconstructed images. No corrections for attenuation, scatter, random coincidences, or dead time were applied.

### D. System Performance

Regarding the performance evaluation of the PET insert, the spatial resolution, sensitivity, count rate, and IQ were inspired by the National Electrical Manufacturers Association (NEMA) NU 4 2008 protocol [9].

#### 1) Spatial Resolution:

A 22Na spherical source with a diameter of 0.25 mm and an activity of 85 kBq (measured in March 2024), was placed at different axial and transaxial positions of the FOV. Initially, the source was positioned at the center within the FOV (cFOV) and displaced along the radial axis to locations at 5, 10, 15, and 25-mm off-center. Then, the source was axially displaced at one-quarter of the axial FOV (qFOV) away from the center and again moved to radial positions at 5, 10, 15, and 25 mm. Data were acquired for 10 min at each position and reconstructed using 10 iterations of the MLEM algorithm in a tradeoff between the spatial resolution and avoiding over-iteration. Although the NEMA NU 4–2008 protocol recommends using the FBP method for spatial resolution assessment, the MLEM algorithm was consistently used for all reconstructions in this study.

The acquisitions were reconstructed using 10 iterations in a tradeoff between the spatial resolution and avoiding over-iteration. The spatial resolution was estimated as the full-width-at-half-maximum (FWHM) of the source profiles in the radial, tangential, and axial directions of the reconstructed images. The source profiles were extracted using AMIDE software [27]. The same analysis was performed both with and without DOI to quantify the relevance of DOI inclusion.

#### 2) Sensitivity:

Sensitivity data was acquired by moving the 22Na source along the axial axis in 2-mm steps across the entire axial FOV (34 positions in total). The acquisitions lasted 8 min at each position. For data analysis, energy windows of 357–664 keV (30% of 511 keV) and 255–766 keV (50% of 511 keV) were used and the sensitivity values were calculated as described in the NEMA protocol.

#### 3) Count Rate Performance:

To evaluate the count rate performance, a cylindrical phantom of high-density polypropylene with dimensions of 25 mm in diameter and 70 mm in length was placed at the center of the FOV. The cylinder has a drilled hole of 3.2-mm diameter hole parallel to the axial axis and at a radial offset of 10 mm in which a fillable silicone capillary filled with 21 MBq of [18F] FDG was inserted. Data was acquired for 13 hours in short acquisitions of 240 s each. Thus, a total of 200 measurements were collected. The values of the True (T), Random + Scatter (R+S) event rates, and the noise equivalent count rate (NECR) were calculated following the NEMA descriptions. Only those events falling within the energy window of 357–664 keV (30% of 511 keV) were considered.

#### 4) Phantom Studies:

The NEMA IQ phantom for small animals was used to assess the image performance of the PET system. The IQ phantom is composed of PMMA material and has internal dimensions of 30 mm in diameter and 50 mm in length. The IQ phantom has two parts: 1) a fillable cylindrical chamber (diameter of 30 mm and 2) a length of 30 mm) with two cold cylindrical regions of 15 mm each. One of the cylinders is filled with distilled water while the other one contains air.

The second part of the phantom consists of a solid section of 20-mm length with five fillable rods (rod diameters 1, 2, 3, 4, and 5 mm) placed at a radial position of 7 mm from the geometrical center of the phantom.

For DAQ, the phantom was filled with 14 MBq of [18F] FDG and the center was placed at the PET cFOV. Data was acquired for 1 hour. The IQ images were reconstructed using 50 iterations of the MLEM algorithm. Following the NEMA protocol [9], the recovery coefficients (RCs), standard deviation (STD), spill-over ratio (SOR), and uniformity values were calculated.

Additionally, a micro-Derenzo phantom was imaged to further study the IQ of the system. This phantom is made of PMMA, composed of several hole pattern sectors with different diameters (1.5, 1.2, 1.0, 0.9, 0.8, and 0.7 mm) and spaced at a distance equal to two times their diameter. The micro-Derenzo phantom was filled with 11 MBq of [^18^F] FDG and placed at the center of the PET system. Data was acquired for 40 min and 150 iterations were used for image reconstruction. To quantify the resolvability of the rods, the Rayleigh criterion was applied [28], [29], [30], [31] as follows: line profiles were drawn in all sectors of the phantom, and then, for each adjacent pair of spots, the valley between them and their peaks were found using parabola fits to extract their intensity. Finally, the valley-to-peak ratios (VPRs) were computed for each pair of spots, in such a way that if the average VPR for the given sector was below the Rayleigh criterion (0.735), the spots of the sector were considered resolvable. Additionally, to determine the resolvability fraction of each sector, it was calculated the proportion of the total number of adjacent spot pairs with a VPR below the 0.735 threshold was calculated.

To give some insights on the capabilities of the PET system to resolve pseudo-preclinical structures, a fillable mouse phantom from Bioemtech was used [32]. It consists of several fillable cavities that simulate the major organs of a mouse and two extra fillable cavities to simulate tumoral lesions. The mouse phantom was filled with 26 MBq of [^18^F] FDG for DAQ. For image reconstruction, 30 MLEM iterations were applied.

### E. Tri-Modal Compatibility Tests

Two different tests were carried out to evaluate the compatibility of the PET insert as a part of a trimodal (PET-MRI-FUS) system: a proof-of-concept experiment using a phantom and an in vivo study.

#### 1) Proof-of-Concept Experiment:

**Fig. 2.**
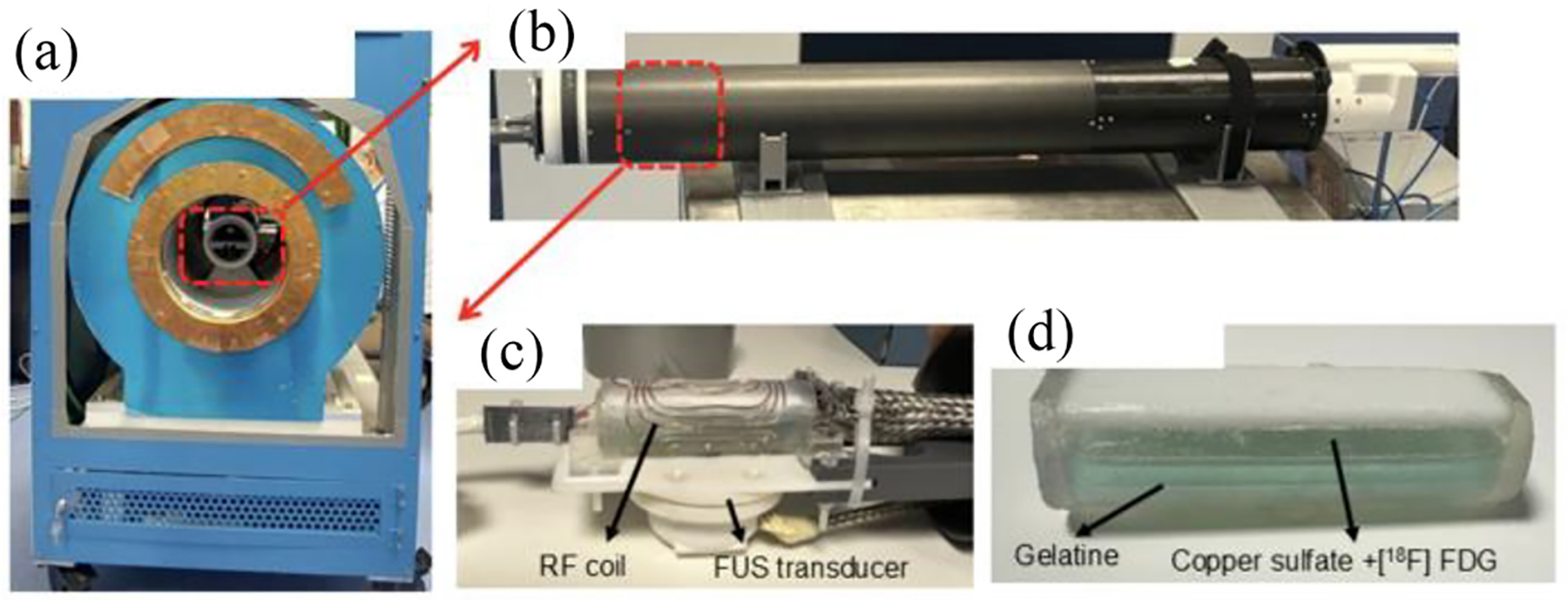
Photograph of the trimodal system: PET (b)–FUS (c)–MRI (a) and of the Phantom (d) used for the study at the hospital La Fe in Valencia. One half of the phantom was filled with solidified gelatine and the other half with copper sulfate and [18F] FDG. The phantom was inside the custom RF coil and placed over the transducer.

We have developed and carried out the experiment at the hospital La Fe in Valencia, Spain. The PET insert was combined with a low-field MRI system and a custom FUS transducer (both developed at the i3M) to test the simultaneous proper operation of the three systems (Fig. 2). The low-field MRI scanner is based on a Halbach cylinder magnet that incorporates 4600 N48 NdFeB cubes of 12-mm side, generating a main magnetic field B0 of 72 mT [33]. The dimensions of this magnet are 53 cm in diameter and 51 cm in axial length with a bore opening of 24 cm which corresponds with the inner diameter of the gradient structure. A specific RF coil was designed with 48-mm axial length and 25 mm in diameter, including 2 lobes of 4 turns. The custom-made FUS transducer, consisted of a function generator (AFG 3100 Series de Tektronix) and an amplifier (1040L de Electronics & Innovation) connected to a focused piezoelectric ceramic of 1MHz coupled to a holographically designed 3-D printed acoustic curved lens [34]. All FUS cables were coated with a thin copper sleeve to prevent MRI cross-interference.

For this experiment, a custom phantom consisting of a cylinder of Elastic 50-A Resin with a top cover of Rigid 4000 (Formlabs) was designed and fabricated. One half of the phantom was filled with a gelatine mixture that melts at temperatures > 30 °C. The other half of the phantom was filled with an aqueous mixture of copper sulfate and ~7 MBq [18F] FDG (Fig. 2). The phantom was introduced inside the trimodal PET-MRI-FUS system. The temperature of the mixture was monitored during the entire process with two thermopar (Sevierville, TN, USA) inserted into the gelatine. The aqueous solution was confined to the top half of the phantom in the initial stage. Next, the center of the phantom was sonicated for 30 min which resulted in heating and phase transition in the gelatine where the aqueous solution has diffused into the gelatine during the final stage of sonication. PET and MRI scans were acquired during every sonication and reconstructed to analyze the deformation caused by this phase transition. To quantify this transition, a profile was extracted from the reconstructed PET images at different stages of the experiment across the center of the cylindrical phantom. MRI scans were acquired using a 2-D-RARE sequence with the following parameters: FoV = 50 × 50 mm^2^, in-plane resolution = 1.25 × 1.25 mm^2^, echo train length (ETL) = 10, echo time (TE) = 10 ms, repetition time (TR) = 200 ms, bandwidth = 10 kHz, and 100 averages, resulting in a total scan time of 1.3 min. The resulting images were denoised using BM3D filtering [35].

#### 2) In Vivo Experiment:

**Fig. 3.**
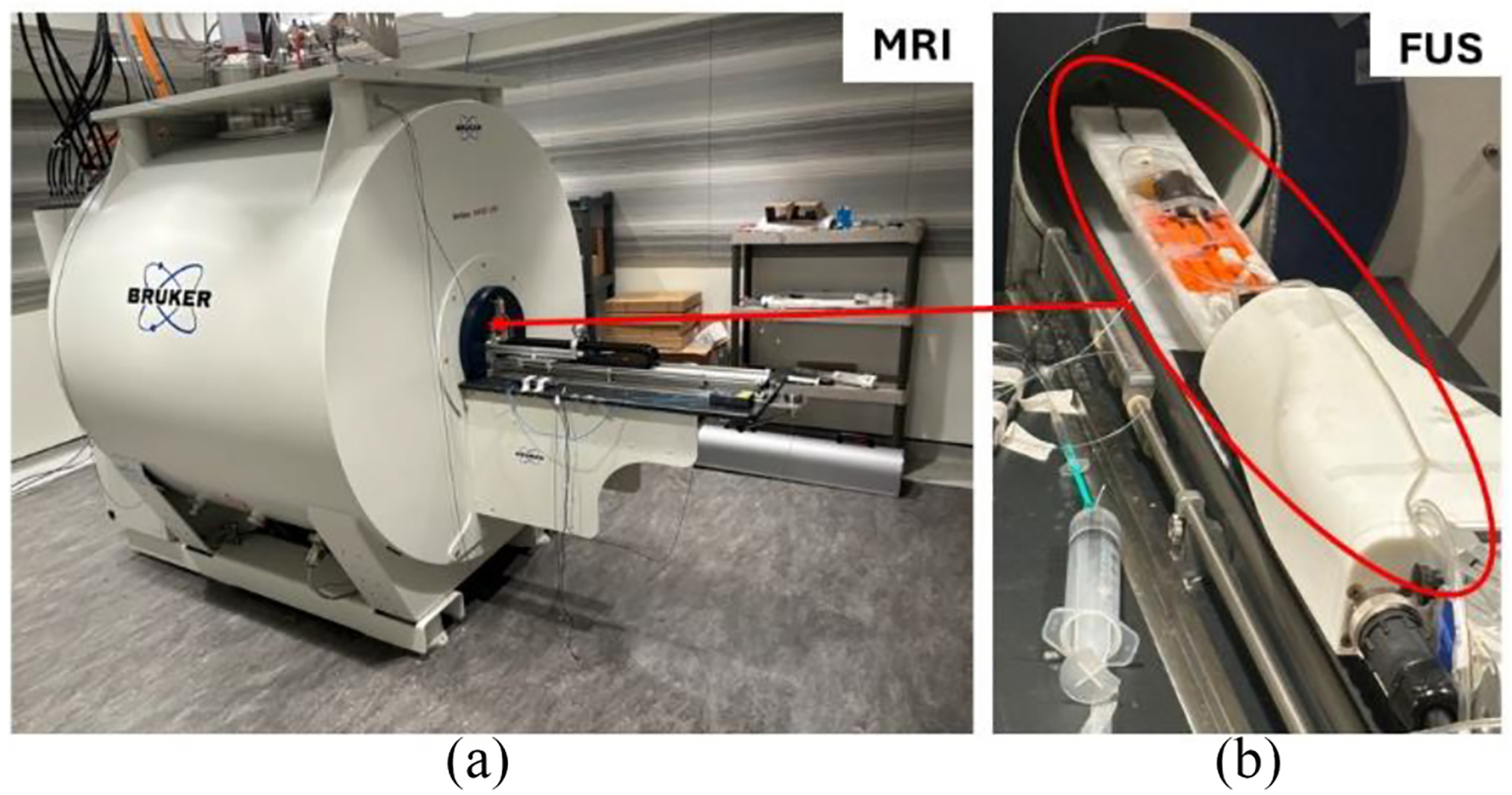
Photograph of the two commercial systems used in the in vivo trimodal test at the University of Virginia (UVa). The mouse was placed supine on the FUS transducer and aligned with the cFOV of the MRI and PET systems.

For the second phase of the trimodal studies, the system was transported to the University of Viriginia (UVa) for in vivo experimentation. The PET insert was used simultaneously with two commercially available systems (Fig. 3), namely: a preclinical high-field MRI scanner (9.4 T, Biospec 94/20 from Bruker) [35], and a preclinical RK-300 FUS Instruments (Toronto, ON, Canada) consisting of a single-element 1.15-MHz spherical transducer. The RK-300 system’s outer diameter was 72 mm and our PET insert was designed to accommodate this [37].

The mouse (wild-type C57Bl6) was intravenously injected with lipid-shelled microbubbles (1×10^5^ /g body mass, synthesized in-house) [38] and 50 ml Gd-DOTA (Dotarem, Guerbet). The MRI scans were acquired using a 3-D T1-weighted Fast Low-Angle Shot (FLASH) sequence. Imaging parameters included a matrix of 150 × 150 × 100, resulting in an isotropic voxel size of 0.285 mm. The TR was 20.8 ms, the TE was 6.4 ms, and a flip angle of 40 ° was used. FUS sonications were applied (1.15 MHz, 0.4 MPa, 0.5% duty cycle, 120 s period) at a single location defined as ROI 1 (as shown in Fig. 11) in the cerebral cortex for 120 s. Cavitation activity of the microbubbles was monitored in real-time during the procedure via passive cavitation detection (PCD). The enhanced region on the MRI was used to calibrate the positioning of the transducer. Then, nine MBq of 64Cu-DOTA (synthesized in-house) along with another dose of microbubbles was injected while sonicating a second region defined as ROI 2 (as shown in Fig. 11). A 5-min-long PET acquisition was started 13 min after the injection of the 64Cu-DOTA. The two DOTA compounds should co-localize with the 64Cu-DOTA being visible in the PET and the Gd-DOTA being visible in the MRI.

All the images acquired for the compatibility studies with both the custom phantom and in vivo samples were reconstructed with 30 MLEM iterations. The PET and MRI scans were co-registered using *π*MOD software [39]. Acoustic emissions (PCD) were processed using a custom algorithm for spectral analysis.

## III. Results

### A. System Performance

#### 1) Spatial Resolution:

**Fig. 4.**
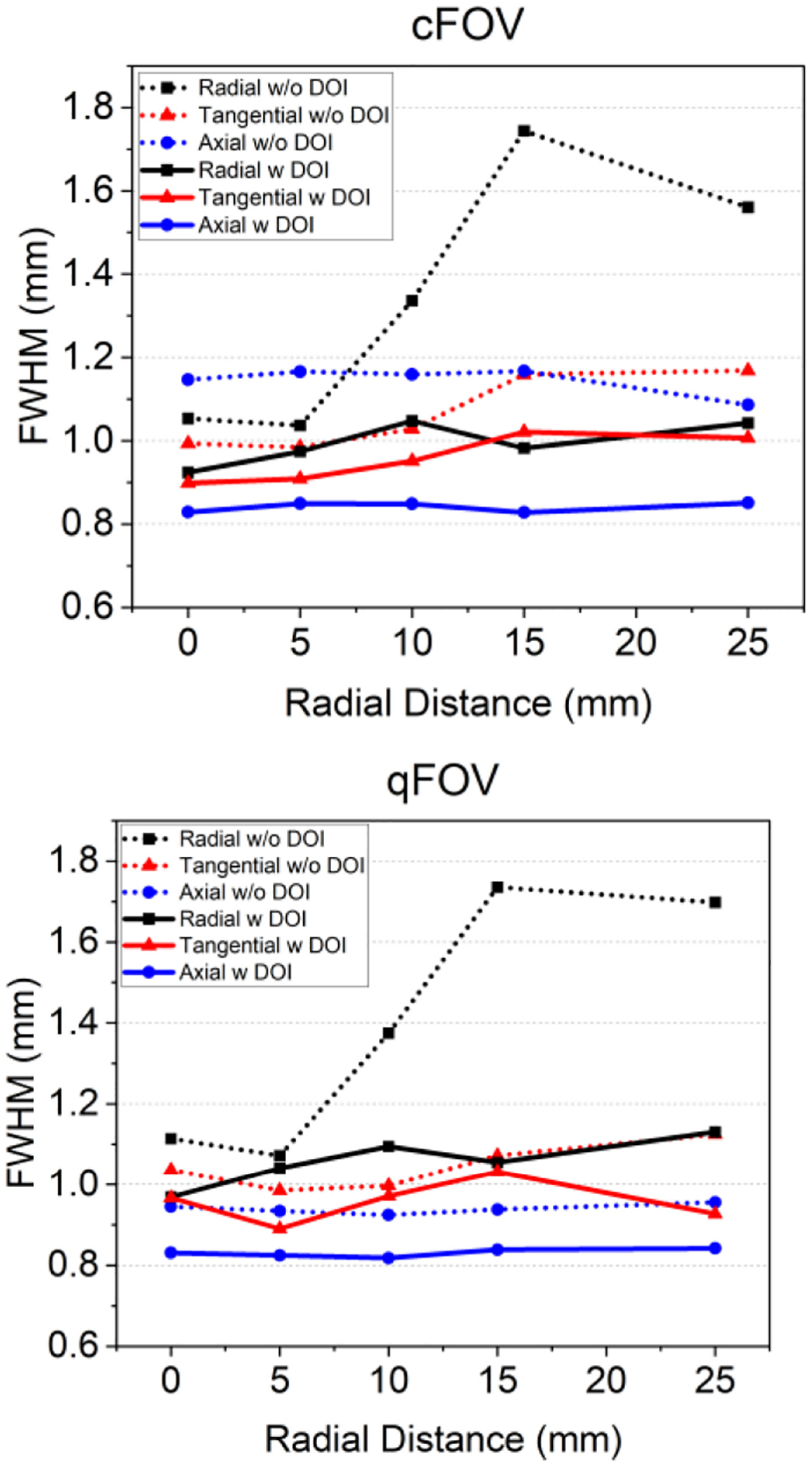
Spatial resolution measured across the radial, tangential, and axial directions when the source was placed at the cFOV (top) and at the qFOV (bottom). The dotted lines report the resolution values without DOI correction, while the solid ones report the same values when, including the DOI information.

The measured spatial resolution as a function of the 22Na source radial position at both the cFOV and the qFOV are shown in Fig. 4. The plots show the resolution values for the radial (black squares), tangential (red triangles), and axial (blue circles) directions. The solid and dotted lines represent the resolution values obtained with and without, including DOI correction, respectively. Table I shows the mean and STD values of the spatial resolution at the two axial positions and spatial directions with and without the DOI correction.

#### 2) Sensitivity:

**Fig. 5.**
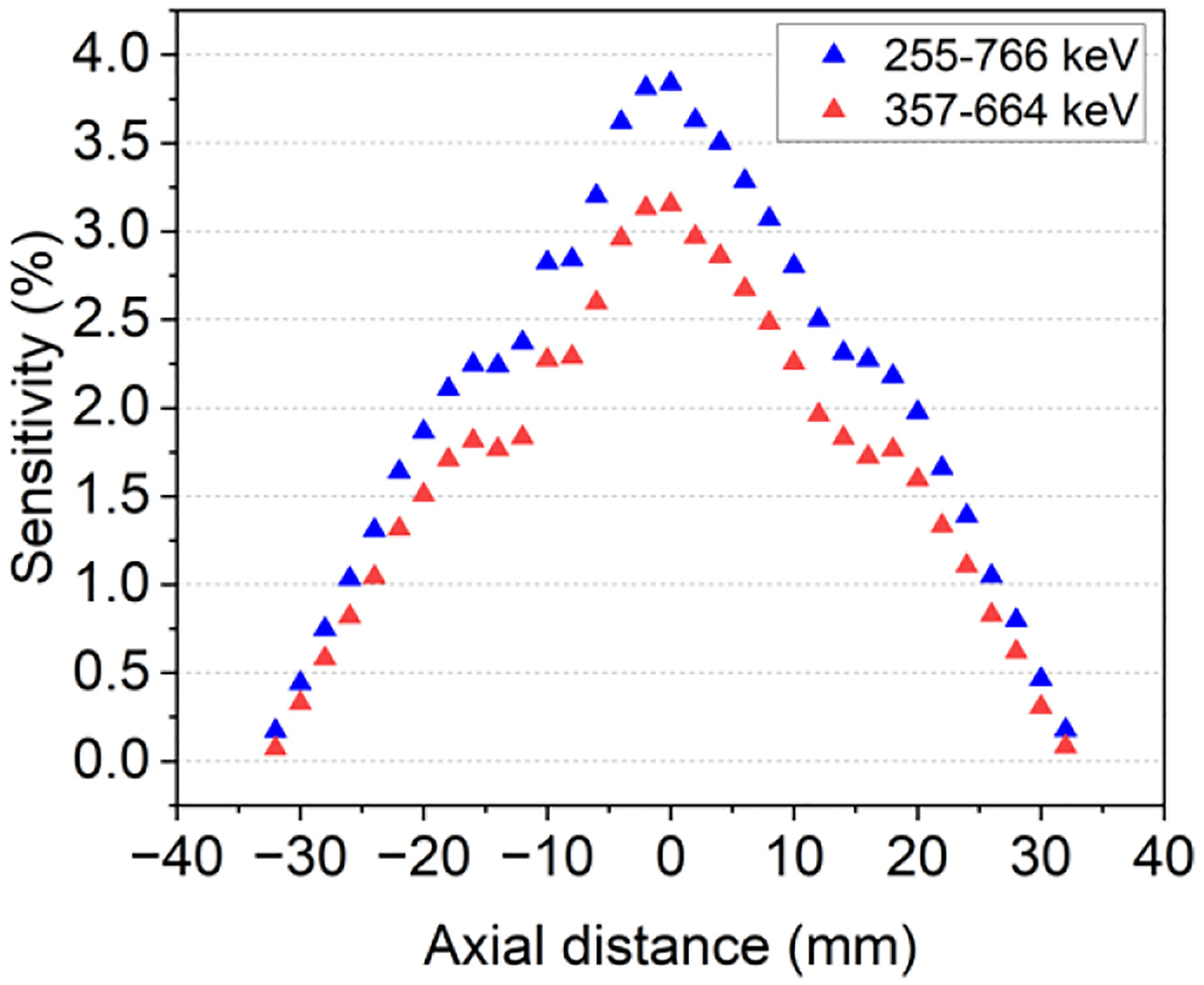
PET system sensitivity profile for energy windows of 357–664 keV (red squares) and 255–766 keV (blue squares).

Fig. 5 depicts the measured sensitivity values across the axial direction of the scanner for the energy windows of 357–664 keV, [511-keV wide window with lower edge at 30% of 511-keV photopeak (red triangles)] and 255–766 keV, [511-keV wide window with lower edge at 50% of 511-keV photopeak (blue triangles)], achieving peak sensitivity values of 3.1% and 3.8%, respectively.

#### 3) Count Rate Performance:

**Fig. 6.**
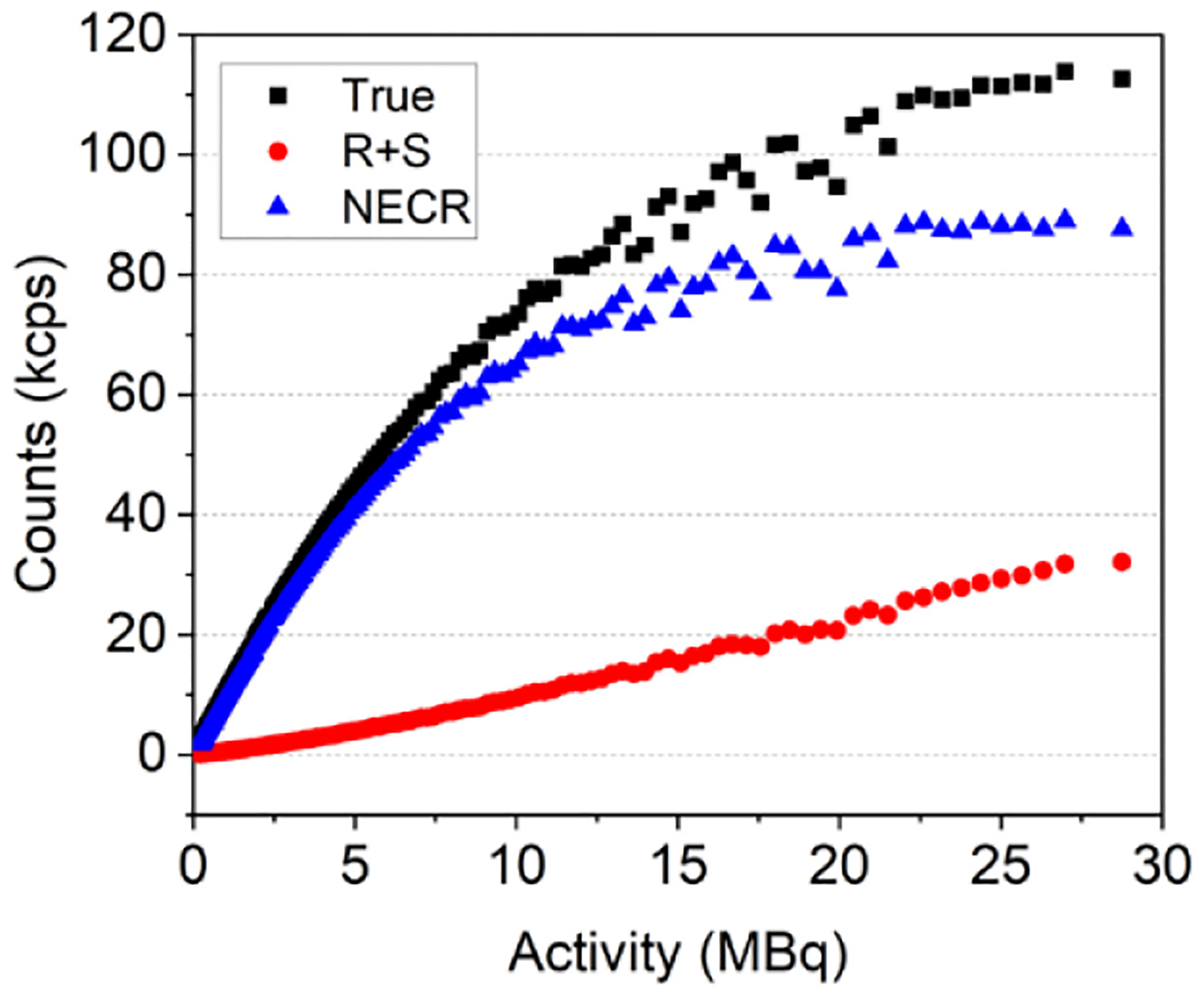
Count rate performance as a function of the total activity for an energy window of 357–664 keV. The red curve represents the R+S events, the blue curve represents the NECR and the black curve represents the T events.

The count rate capabilities of the PET insert are reported in Fig. 6. The T, R+S, and NECR curves are represented in black, red, and blue colors, respectively. The NECR peak reached of approximately 80 kcps was reached for an activity in the range of 17–25 MBq (activity concentration: 283–383 MBq/ml).

#### 4) Phantom Studies:

**Fig. 7.**
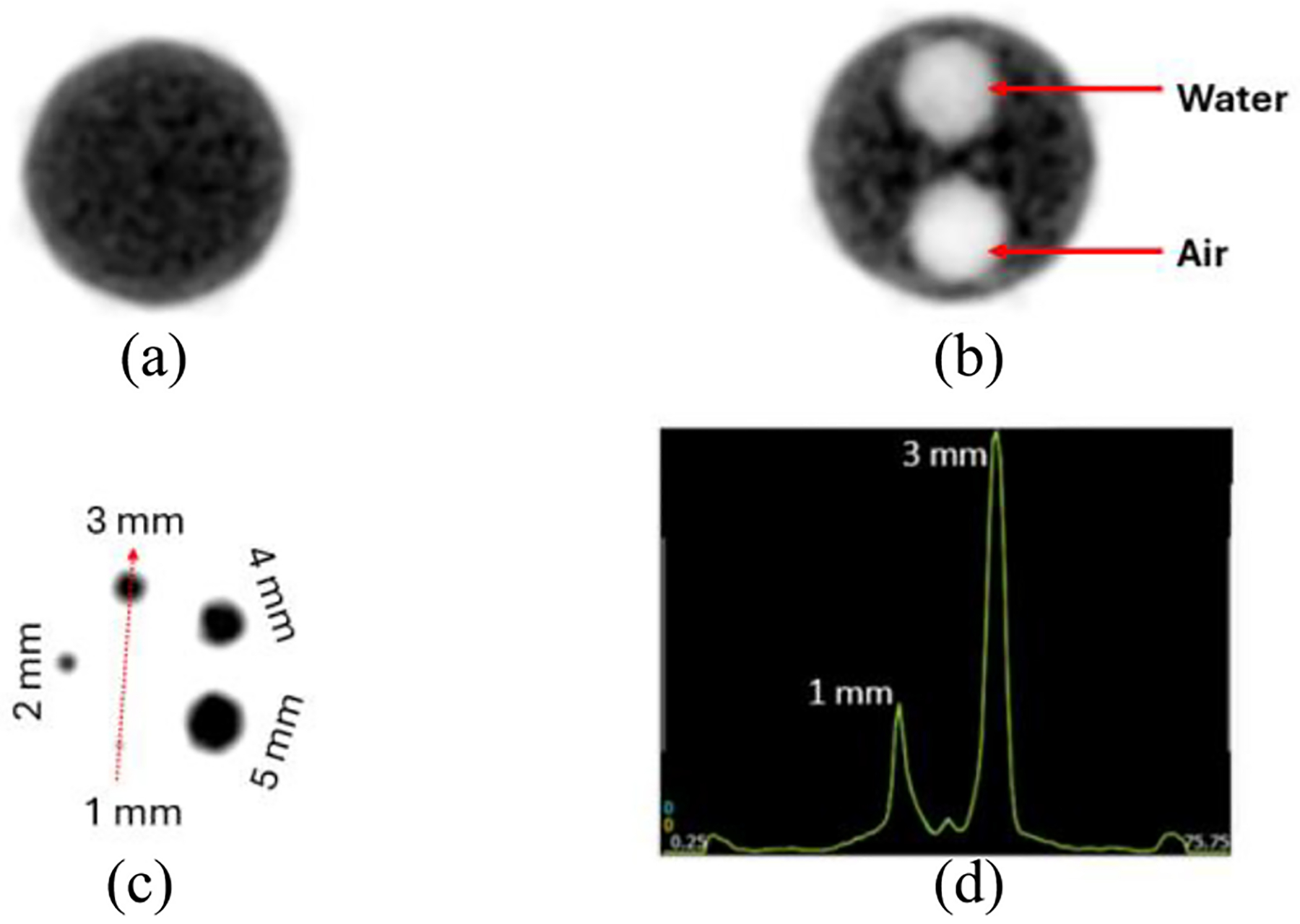
Transaxial view of the three different regions of the IQ phantom. (a) Uniform region. (b) Cold chambers region. (c) Hot rods region. (d) Profile extracted across the 1 and 3-mm rods crossed by the red arrow in Fig. 7(c).

Fig. 7 shows the reconstructed images of the NEMA IQ phantom. Specifically, panels 7(a)–(c) show the different regions of the phantom, namely: uniform region, cold chambers (air and water) and hot rods region. Fig. 7(d) shows the image profile along the 1 and 3-mm rods that cross the red arrow in Fig. 7(c).

Table II displays the RCs for the hot rods region of the NEMA IQ phantom. RCs exceed 0.8 for rods with diameters of 3 mm or more. The SOR values for the air- and water-filled cold chambers reached values of 0.11 and 0.22, respectively.

**Fig. 8.**
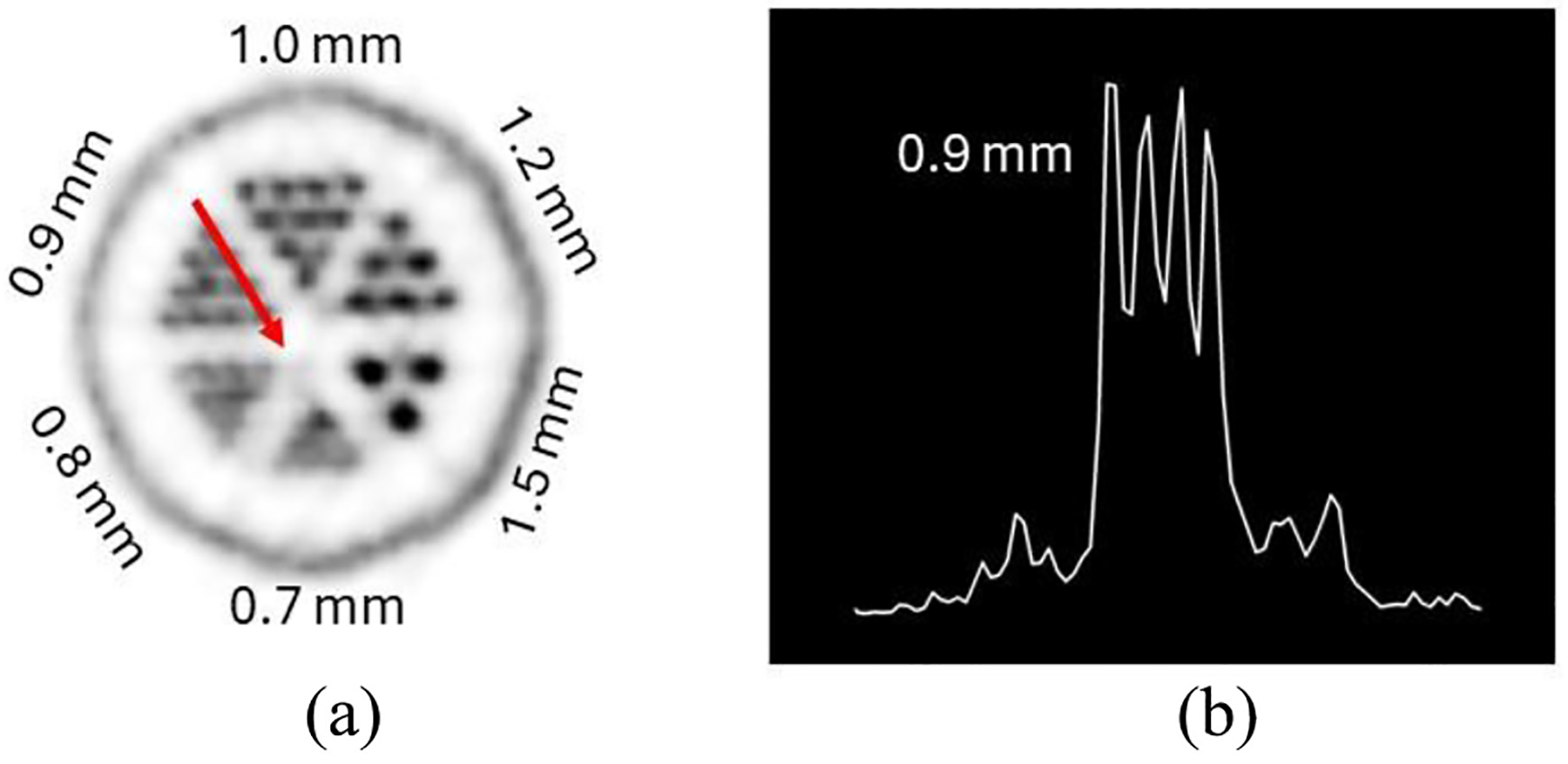
Reconstruction of the micro-Derenzo phantom. (a) Transaxial view of the rod’s region and the red arrow that represents the extracted profile. (b) Profile extracted over the 0.9-mm rod sector.

Regarding the study with the micro-Derenzo phantom, Fig. 8(a) depicts the transaxial view of this phantom, showing the different rod sectors. The profile over the 0.9-mm rods (crossed by the red arrow in Fig. 8(a) has been extracted and is reported in Fig. 8(b).

Table III shows the values for the average VPR, STD VPR and resolvability for the different micro-Derenzo rod sectors. Following the Rayleigh criterion, the rod sectors with diameters of 0.9 mm and bigger are considered resolvable, as they have an average VPR below 0.735. Moreover, for the 1.5 and 1.2-mm sectors 100% of the rods were resolved.

**Fig. 9.**
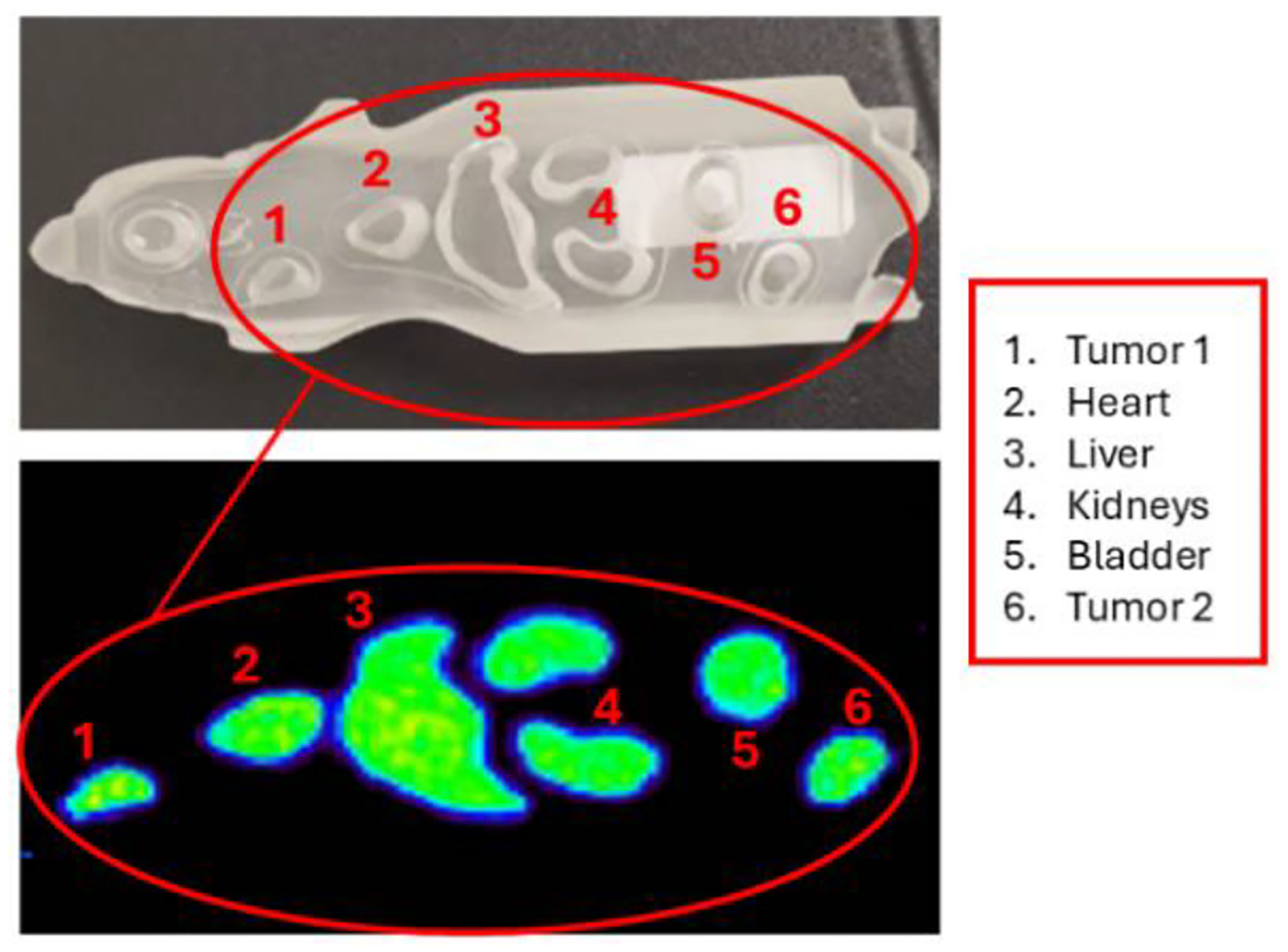
Reconstructed image for the lower region of the mouse phantom showing the fillable cavities: two tumors, heart, liver, kidneys, and bladder.

The reconstructed image of the mouse fillable phantom is shown in Fig. 9. The phantom was larger than the axial coverage of the PET insert, and thus, only about 80% of the phantom (area inside the red circle) was imaged. The cavities representing two tumors, the heart, liver, kidney, and bladder of the mouse can be observed.

### B. Tri-Modal Compatibility Tests

#### 1) Proof-of-Concept Experiment:

**Fig. 10.**
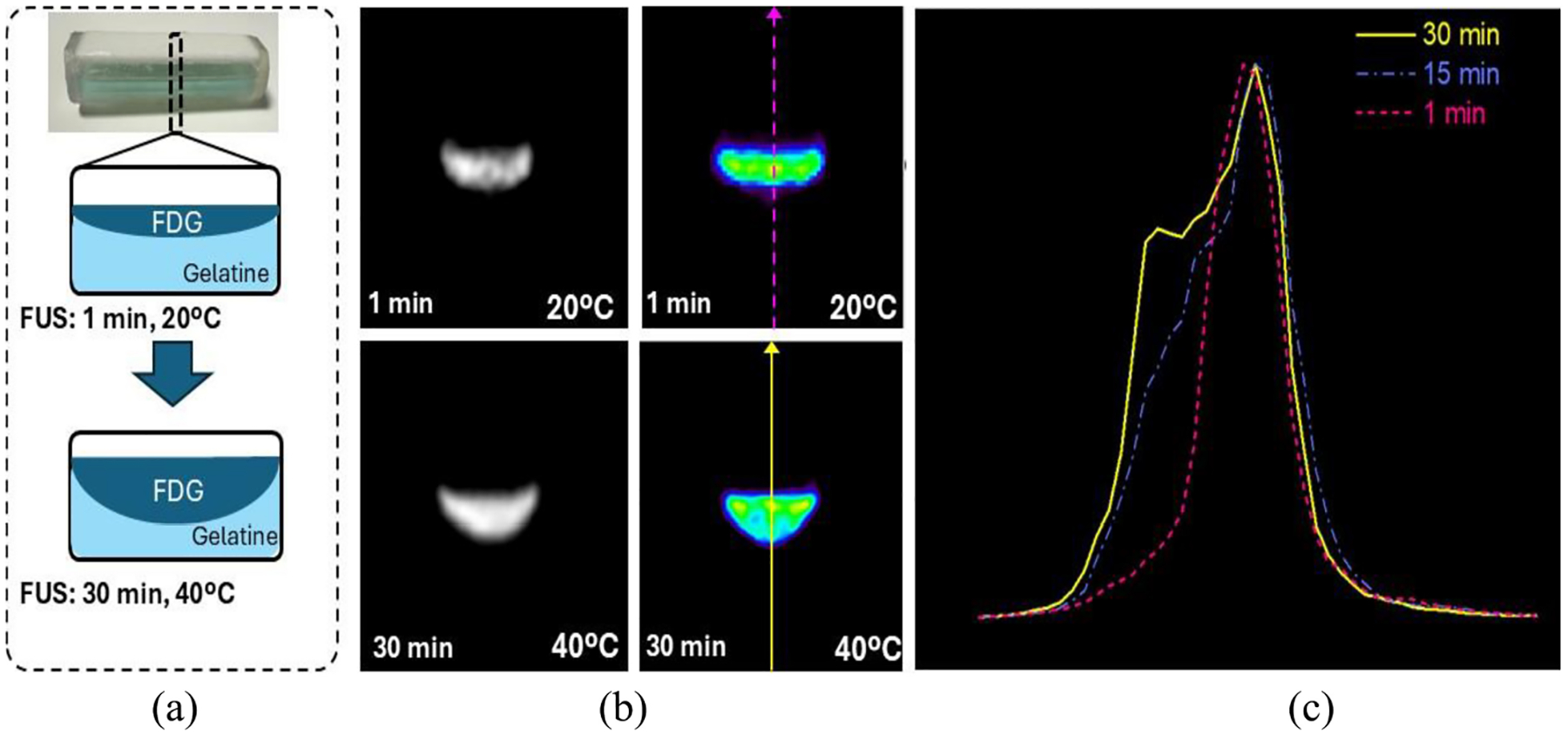
Reconstructed images from the simultaneous DAQ with the PET insert, low-field MRI, and in-house FUS device. (a) Transversal view of the phantom composition at two time points: before (1 min, 20 °C) and after (30 min, 40 °C) FUS application. (b) MRI images of the phantom at those two time points. (c) Corresponding PET reconstructions for the same time points. (d) Activity profiles extracted from the PET images at 1, 15, and 30 min, showing the mixture of radiotracer with the gelatine.

Fig. 10 summarizes the reconstructed images when simultaneously acquiring data with the developed PET insert and the low-field MRI while sonicating the cylindrical phantom with an in-house FUS device. Fig. 10(a) shows a transverse view of the phantom, highlighting the composition of the phantom and the change in its internal components at different times of the FUS sonication procedure.

The reconstructed MRI and PET images at two different times are shown in Fig. 10(b) and (c), respectively: before significant heating (1 min, 20 °C) and after prolonged FUS application (30 min, 40 °C). Fig. 10(d) presents the activity profiles extracted from the PET images at 1, 15, and 30 min, showing the evolution of the radiotracer distribution over time, and specifically how it diffuses when the gelatine is melted by the FUS device.

#### 2) In vivo Experiment:

**Fig. 11.**
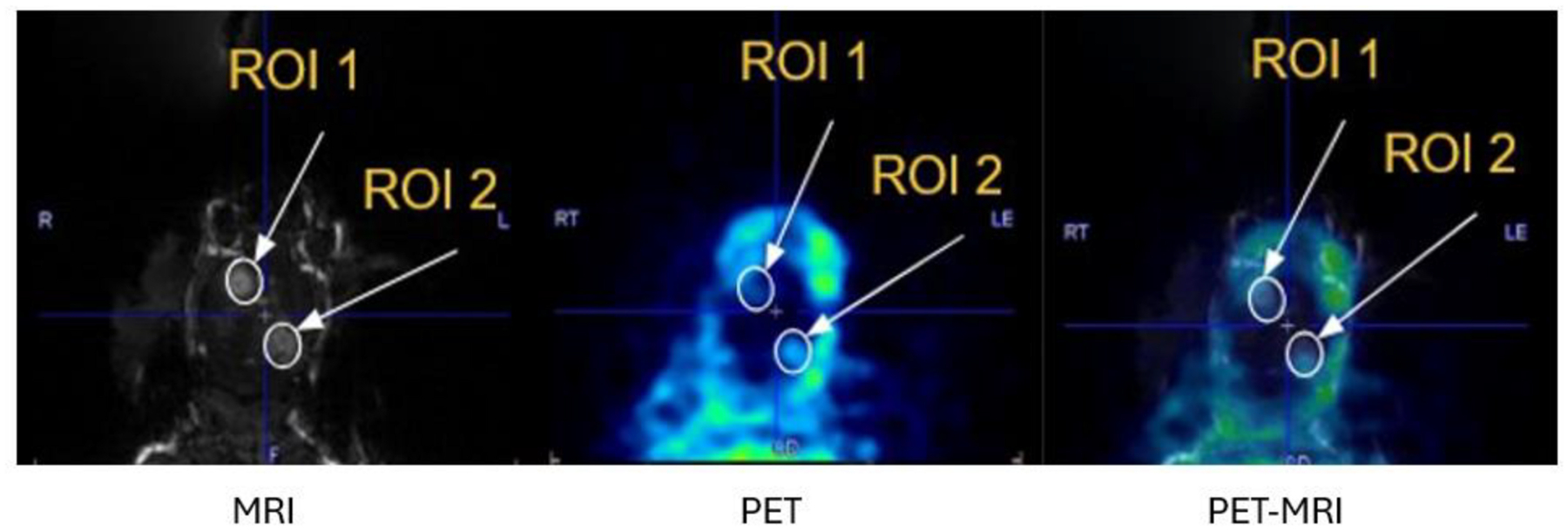
Images of the mouse in vivo experiment. Left: Reconstructed MRI image acquired after FUS sonication of two locations in the brain. The resulting two areas of Gd-DOTA enhancement were used to define two regions of interest labeled ROI 1 and ROI 2. Middle: Reconstructed PET image acquired following the second sonication. Co-localization between the point of 64Cu-DOTA enhancement and the MRI-defined ROI 2 demonstrates the good co-alignment of the integrated MRI and PET components. Right: The co-registered PET and MRI images.

The left image in Fig. 11 is a slice from the T1-weighted MRI scan acquired after both FUS sonications. Two regions of interest labeled ROI 1 and ROI 2 were defined based on the enhanced regions in the MR images using ITK-SNAP [40]. For comparison with unsonicated areas of the brain, two regions of interest (not shown) were also created on the contralateral, unsonicated side of the brain by reflecting each of the sonicated ROI perimeter voxels across the midline sagittal plane of the mouse. The four regions of interest defined using the MR images were then transferred to the co-registered PET images to determine if 64Cu-DOTA accumulated in the same regions of the brain. The average PET voxel value in ROI 2 was 43% higher than that in the contralateral side while the average PET voxel value in ROI 1 was 37% higher than that in the contralateral side. The slightly lower activity in ROI 1 was not unexpected as some time had elapsed since the first sonication before the 64Cu-DOTA was injected allowing the BBB time to partially close.

## IV. Discussion

The present study reports for the first time the direct integration of an FUS device with a PET/MR design. In particular, the manuscript focuses on the evaluation of a small-animal PET insert-based monolithic LYSO: Ce crystals with DOI capabilities. The PET was designed to be MRI- and FUS-compatible and was evaluated following the NEMA NU-4 protocol to gain insight regarding its performance. The performance of the PET insert has been compared with four inserts of similar characteristics, namely: SimPET/M7 [10], I-402 [11], MADPET4 [12], and Si-198 [13].

Regarding the spatial resolution study, submillimeter spatial resolution was observed across the entire FOV for cFOV and qFOV positions, as shown in Table I. Notably, the inclusion of DOI correction significantly improved the resolution, especially in the radial direction, where the resolution improved from 1.36 ± 0.31 mm (without DOI) to 0.99 ± 0.05 mm (with DOI). This is a key strength of our system because in preclinical PET scanners, due to the small FOV, parallax errors highly impact the spatial resolution, especially at the edges of the FOV. The spatial resolution values achieved at the cFOV are comparable with the SimPET/M7 and Si-198 systems (Tables IV and V). Analyzing the spatial resolution at the edges of the FOV, better values were obtained with the proposed system compared to pixelated-based systems without DOI capabilities.

The sensitivity profile follows the expected behavior (Fig. 5), with a maximum value at the cFOV comparable to the SimPET/M7, higher than the MADPET4 and lower than the I-402 and Si-198 due to their higher axial coverage. The PET insert exhibits a lower NECR peak compared to the other systems. This difference may also arise from the thinner scintillators used and the shorter axial length of this prototype when compared to other systems. Notice that the thickness of the scintillators in the current design is only 8 mm, which allows the integration with the FUS and the MRI. In the IQ phantom, all rods were resolved, matching the submillimeter spatial resolution achieved with the 22Na spherical source. The RCs, SOR and uniformity values found with the IQ phantom are comparable with the state-of-the-art systems. Moreover, the reconstructed images from the micro-Derenzo phantom were evaluated, demonstrated clear resolution of rods down to a size of 0.9 mm (as shown in Table III).

Our proof-of-concept studies have demonstrated the ability of the PET insert to operate simultaneously as part of an in-house trimodal PET-low-field MRI-FUS system. Simultaneous PET and MRI scans were successfully achieved during FUS sonication. The profiles extracted from these images illustrated the diffusion of a copper sulfate and [18F] FDG mixture inside a gelatine phantom, demonstrating the potential of this platform to monitor biological processes modulated by FUS. These tests confirmed the mechanical and electromagnetic compatibility of the three modalities.

In the preliminary in vivo experiment, compatibility was further validated using a high-field 9.4T MRI and a commercial FUS system. PET and MRI scans acquired during FUS sonication of naïve murine brain were successfully obtained, reconstructed, and co-registered - confirming the ability of the trimodal platform to perform concurrent imaging during a FUS sonication without significant signal degradation or artifacts.

We were also able to demonstrate that we could use imaging to show where the blood-brain barrier had been opened by FUS with microbubbles using either Gd-DOTA enhanced MRI or 64Cu-DOTA PET. Both modalities demonstrated measurable changes in contrast agent concentration across the BBB in regions of the brain that had been opened by FUS. In the future, our goal is to use PET imaging to follow the uptake of radiolabeled large-molecule therapeutic agents or to use the PET signal to guide the location where FUS energy is applied. This is the first device of its kind that can be used for these types of studies. To enable these applications, further improvements in reconstruction software are planned, including the implementation of dead-time and other quantitative corrections to ensure accurate activity estimation in dynamic or high-activity studies.

The integration of PET, MRI, and FUS into a single platform represents a significant advancement in preclinical image-guided therapy. This trimodal system has the potential to enable innovative strides across numerous areas of FUS research, including BBB opening for drug delivery, neuromodulation, immuno-modulation, sonodynamic therapy and more [41], [42], [43]. The success of the compatibility tests reported herein opens the possibility of applying this novel technological platform to advanced biological and pharmacological research. It also lays the groundwork for extending this technology into human-sized systems that could be used in clinical trials.

## V. Conclusion

The integration of PET, MRI, and FUS into a single platform represents an innovative step in preclinical imaging. Previous approaches have relied on sequential or dual-modality systems, which limited the ability to simultaneously acquire complementary functional, anatomical, and therapeutic information.

In this work, a preclinical PET insert based on 16 monolithic LYSO:Ce crystals was successfully designed, constructed, and evaluated following the NEMA NU 4 2008 protocol for preclinical systems. Additional tests were carried out in concert with MRI and FUS systems since the PET insert was developed for the eventual realization of a first-in-class trimodal PET/MRI-guided FUS system.

The PET insert demonstrated high performance, achieving submillimeter resolution, a state-of-the-art sensitivity peak, and excellent imaging performance, all of which were on par with other preclinical PET inserts—highlighting its potential for high-precision imaging in small animal studies. The results of the compatibility assessments with MRI and FUS systems demonstrated the ability of the PET insert to work simultaneously in a trimodal setting, achieving, to the best of our knowledge, the first hybrid PET-MRI imaging acquired during FUS sonications.

Future studies will focus on optimizing the PET system performance by adding quantification features, enhancing image corrections and a pseudo-automatic PET-MRI co-registration algorithm, aiming to explore biological processes in greater detail and enable advanced in vivo experiments.

## Figures and Tables

**TABLE I:** Mean Spatial Resolution (in mm) With and Without DOI Correction

Axial Position	Radial		Tangential		Axial	
	W/O DOI	wDOI	W/O DOI	wDOI	W/O DOI	wDOI
**cFOV**	1.36 ± 0.31	0.99 ± 0.05	1.07 ± 0.09	0.95 ± 0.06	1.15 ± 0.03	0.84 ± 0.01
**qFOV**	1.39 ± 0.31	1.06 ± 0.06	1.04 ± 0.06	0.96 ± 0.04	0.94±0.01	0.84 ± 0.01

**TABLE II:** RCS for the Different Rods Using the NEMA IQ Phantom

Ø Rod (mm)	1	2	3	4	5
**RC**	0.31	0.68	0.81	0.85	0.89

**TABLE III:** Values of the Rayleigh Criterion for the Rod Sectors of the Micro-Derenzo Phantom

Ø Rod (mm)	1.5	1.2	1.0	0.9	0.8
**Average VPR**	0.35	0.47	0.62	0.67	0.85
**STD VPR**	0.03	0.11	0.16	0.15	0.13
**Resolvability (%)**	100	100	78	67	22

**TABLE IV:** Comparison of Uniformity, RCs AND SORs of the Designed Insert PET (Highlighted) With State-of-the-Art Preclinical PET Inserts

	Type	Uniformity (%)	RCs (1–5 mm)	SOR air (%)	SOR water (%)
**Our insert**	Monolithic	4.8	0.31–0.89	11	22
**SimPET** [10]	Pixelated	4.4	0.17–0.90	13	15
**I-402** [11]	Pixelated	4.3	0.28–0.90	35	24
**MADPET4** [12]	Pixelated	8.3	0.12–0.95	15	24
**Si-198** [13]	Monolithic	6.5	0.14–0.94	12	22

**TABLE V:** Comparison of Spatial Resolution, Peak Sensitivity and NECR Peak of the Designed Insert PET (Highlighted) With State-of-the-Art PreclinicaL PET Inserts. Sensitivity Refers to Peak Sensitivity and NECR Refers to NECR Peak

	Type / Thickness (mm)	Axial FOV (mm)	Inner diameter (mm)	Spatial Resolution (mm)		Sensitivity (%)	NECR (kcps @MBq)
				cFOV	15 mm cFOV		
Our insert	Monolithic (8)	67	72	0.9	0.9	3.8	80 @19
SimPET [10]	Pixelated (10)	55	63	0.9	1.5	4.2	151 @38
I-402 [11]	Pixelated (10)	98	60	1.5*	1.9*	10.15	175 @17
MADPET4 [12]	Pixelated (20)	20	88	1.5**	1.8**	0.7	103 @29
Si-198 [13]	Monolithic (10)	150	114	0.9	0.9	11	486 @23

* Reconstructed with 2D FBP

** Reconstructed with 3D FBP
